# Exploring the alpha‐gliadin locus: the 33‐mer peptide with six overlapping coeliac disease epitopes in *Triticum aestivum* is derived from a subgroup of *Aegilops tauschii*

**DOI:** 10.1111/tpj.15147

**Published:** 2021-02-19

**Authors:** Jan G. Schaart, Elma M. J. Salentijn, Svetlana V. Goryunova, Charity Chidzanga, Danny G. Esselink, Nick Gosman, Alison R. Bentley, Luud J. W. J. Gilissen, Marinus J. M. Smulders

**Affiliations:** ^1^ Plant Breeding Wageningen University and Research Droevendaalsesteeg 1 NL‐6708 PB Wageningen the Netherlands; ^2^ The John Bingham Laboratory NIAB 93 Lawrence Weaver Road Cambridge CB3 0LE UK; ^3^ Bioscience Wageningen University and Research Droevendaalsesteeg 1 NL‐6708 PB Wageningen the Netherlands; ^4^ Allergy Consortium Wageningen Droevendaalsesteeg 1 NL‐6708 PB Wageningen the Netherlands; ^5^ Present address: FSBSI Lorch Potato Research Institute Kraskovo 140051 Russia; ^6^ Present address: Institute of General Genetics Russian Academy of Science Moscow 119333 Russia; ^7^ Present address: University of Adelaide School of Agriculture, Food and Wine Waite Campus Urrbrae South Australia 5064 Australia; ^8^ Present address: Gosman Associates Ag‐Biotech Consulting the Street Bressingham, Diss IP22 2BL UK; ^9^ Present address: International Maize and Wheat Improvement Center (CIMMYT) Texcoco Mexico

**Keywords:** D genome, gluten, alpha‐gliadin, re‐synthesised bread wheat, synthetic hexaploid wheat, SHW, T‐cell epitope, coeliac disease, *Aegilops tauschii*, *Triticum aestivum*

## Abstract

Most alpha‐gliadin genes of the *Gli‐D2* locus on the D genome of hexaploid bread wheat (*Triticum aestivum*) encode for proteins with epitopes that can trigger coeliac disease (CD), and several contain a 33‐mer peptide with six partly overlapping copies of three epitopes, which is regarded as a remarkably potent T‐cell stimulator. To increase genetic diversity in the D genome, synthetic hexaploid wheat lines are being made by hybridising accessions of *Triticum turgidum* (AB genome) and *Aegilops tauschii* (the progenitor of the D genome). The diversity of alpha‐gliadins in *A. tauschii* has not been studied extensively. We analysed the alpha‐gliadin transcriptome of 51 *A. tauschii* accessions representative of the diversity in *A. tauschii*. We extracted RNA from developing seeds and performed 454 amplicon sequencing of the first part of the alpha‐gliadin genes. The expression profile of allelic variants of the alpha‐gliadins was different between accessions, and also between accessions of the Western and Eastern clades of *A. tauschii*. Generally, both clades expressed many allelic variants not found in bread wheat. In contrast to earlier studies, we detected the 33‐mer peptide in some *A. tauschii* accessions, indicating that it was introduced along with the D genome into bread wheat. In these accessions, transcripts with the 33‐mer peptide were present at lower frequencies than in bread wheat varieties. In most *A. tauschii* accessions, however, the alpha‐gliadins do not contain the epitope, and this may be exploited, through synthetic hexaploid wheats, to breed bread wheat varieties with fewer or no coeliac disease epitopes.

## Significance Statement

We sequenced the expressed alpha‐gliadins in grains of *Aegilops tauschii* across its range. Some accessions contained the 33‐mer peptide with six overlapping coeliac disease epitopes, indicating that this was introduced into bread wheat from *A. tauschii*. We also found a large variation in the occurrence of coeliac disease epitopes, including accessions with small numbers of epitopes, suitable as basis for breeding bread wheat varieties with fewer or no coeliac disease epitopes.

## INTRODUCTION

The genome of allohexaploid (2*n* = 6*x* = 42) bread wheat (*Triticum aestivum*) is composed of three subgenomes (A, B and D). It originated from hybridisation between allotetraploid *Triticum turgidum* (AB) and the diploid species *Aegilops tauschii* (D) around 8000 years ago (Nesbitt and Samuel, 1996). This hybridisation probably took place in agricultural fields, as bread wheat does not exist as a wild species. The genetic variation in the D genome of bread wheat is much lower than that present in the A and B genomes (Dubcovsky and Dvorak, 2007). This suggests that the hybridisation event involved only a small subset of *A. tauschii* genotypes, resulting in a strong genetic bottleneck (Dvorak *et al*., 1998). The notion of a hybridisation bottleneck is supported by four recent population genetics studies that show high levels of genetic diversity among genebank accessions of wild *A. tauschii* accessions sampled across the species range, based on the 10K Infinium single nucleotide polymorphism (SNP) array (Wang *et al*., 2013), on 15 D genome‐specific microsatellite markers (Jones *et al*., 2013), on an ultra‐high‐density 817K Affymetrix array (Winfield *et al*., 2016) and on 13 135 SNPs from genotyping‐by‐sequencing data (Singh *et al*., 2019). These studies confirmed the main division between Western (Iran, Turkey, Caucasus) and Eastern (Central Asia) *A*. *tauschii* accessions based on nuclear DNA (e.g. Lubbers *et al*., 1991; Dvorak *et al*., 1998; Pestsova *et al*., 2000), on chloroplast data (Dudnikov, 2012) or both (Mizuno *et al*., 2010). In addition, Mizuno *et al*. (2010), Wang *et al*. (2013), Jones *et al*. (2013) and Matsuoka *et al*. (2015) further distinguished several subgroups within the two regions. Interestingly, previous studies pinpoint a specific *A. tauschii* subgroup located south and southwest of the Caspian Sea in northern Iran as the main source of the bread wheat D genome. This subgroup was coded as 2E by Wang *et al*. (2013) (and S‐2 in Gill’s (2013) commentary on that paper) and as IIID/IIIE by Jones *et al*. (2013).

Wheat consumption may cause allergies and intolerances in some people. The prevalence of immunoglobulin E‐mediated allergy to wheat (and to cereals in general) is low (Gilissen *et al*., 2014), but 1–2% of the people can become intolerant to gluten proteins from wheat, rye and barley and may develop coeliac disease (CD), a chronic inflammation of the small intestine. This inflammation leads to a variety of symptoms and therefore most patients remain undiagnosed (Scherf *et al*., 2020). Coeliac disease is one of the best understood food intolerances with regard to human immunology and T‐cell specificity (Tye‐Din *et al*., 2010; Petersen *et al*., 2014, 2016; Jabri and Sollid, 2017; Sollid, 2017; Dahal‐Koirala *et al*., 2019; Scherf *et al*., 2020; Sollid *et al*., 2020). However, no treatment exists and the only way to prevent CD symptoms is to follow a gluten‐free (GF) diet, requiring complete exclusion of wheat, barley and rye. This is very difficult to adhere to, as gluten (especially from wheat) is added to a broad range of food products (Gallagher *et al*., 2004; Jouanin *et al*., 2018) due to its viscoelastic and binding properties (Atchison *et al*., 2010; Shewry, 2019).

Alpha‐gliadins, along with omega‐gliadins and gamma‐gliadins, are the most important source of immunogenic peptides triggering the T‐cell reaction in CD patients. Alpha‐gliadins are a multigene family, encoded by the *Gli‐2* locus on the short arm of the group 6 chromosomes of bread wheat (Anderson *et al*., 2006), which includes intact as well as pseudogenes (van Herpen *et al*., 2006; Huo *et al*., 2019). Zhang *et al*. (2015) cloned and sequenced 23 alpha‐gliadins in *Triticum urartu* (A genome), of which 12 were intact genes. Huo *et al*. (2017) found 12 alpha‐gliadin genes clustered within a 550‐kb region, of which five were pseudogenes. Noma *et al*. (2016) sequenced alpha‐gliadins from the hexaploid variety Chinese Spring (CS), and found 90 genes, of which 50 were intact (16 on the A genome, 16 on the B genome and 18 on the D genome). Huo *et al*. (2018) re‐examined the CS set of alpha‐gliadin genes in the genome sequence of this variety (The International Wheat Genome Sequencing Consortium [IWGSC], 2018) using additional long‐read sequences, and annotated 47 alpha‐gliadin genes (26 on the A genome, 11 on the B genome, 10 on the D genome), of which 28 were expressed. Noma *et al*. (2019) used gene‐specific primers and found that 26 alpha‐gliadins were expressed in the developing endosperm of CS. Note that the number of genes varies between wheat varieties, and that expression levels vary among genes and between varieties (Shewry and Lookhart, 2003; Salentijn *et al*., 2009; Noma *et al*., 2019; Jouanin *et al*., 2020a).

A large amount of genetic variation exists for the presence of T‐cell‐stimulatory sequences among wheat species and accessions (Spaenij‐Dekking *et al*., 2005; Molberg *et al*., 2005; Van den Broeck *et al*., 2010a; Shewry and Tatham, 2016). Some alpha‐gliadin genes encode for proteins which contain more CD epitopes than others. The observed variation among the genes is genome specific, and the alpha‐gliadins from the D genome have the highest CD‐immunogenic potential (van Herpen *et al*., 2006; Salentijn *et al*., 2009; Mitea *et al*., 2010; Jouanin *et al*., 2019) as most of these genes contain at least one copy of each of three different DQ2 epitopes, of which glia‐α1 is the one to which most CD patients react. Several *Gli‐D2* gliadins also contain a 33‐mer peptide with six partly overlapping copies of the T‐cell epitopes DQ2.5‐Glia‐α1a, DQ2.5‐Glia‐α1b and DQ2.5‐Glia‐α2; this is regarded as a remarkably potent T‐cell stimulator (Qiao *et al*., 2004). In addition, many of the alpha‐gliadins contain the p31‐43 epitope, which is involved in inducing the innate immune response that initiates the development of CD (Maiuri *et al*., 1996, 2003). This epitope is present in alpha‐gliadins from all three subgenomes. Screening studies suggest that there are no modern bread wheats that do not contain several alpha‐gliadin proteins encoded by the D genome locus with several CD epitopes each (Van den Broeck *et al*., 2010a,b; Jouanin *et al*., 2018).

As a means to introduce new genetic variation into the D genome of bread wheat, novel hexaploid wheats are being synthesised by hybridising *T. turgidum* subsp. *durum* with *A. tauschii* accessions, followed by chemical chromosome doubling. These so‐called re‐synthesised bread wheats or synthetic hexaploid wheats (SHWs) are made with the goal of introducing new functional trait diversity into modern germplasm (Kishii, 2019), notably disease resistance genes (Mujeeb‐Kazi *et al*., 1996; Das *et al*., 2016; Szabo‐Hever *et al*., 2018; Kishii *et al*., 2019; Mohler *et al*., 2020) but also, for example, a higher yield (Hao *et al*., 2019). The International Maize and Wheat Improvement Center (CIMMYT), Texcoco, Mexico has been developing many synthetic hexaploid wheats since the 1960s (Gordon *et al*., 2019), but several other programmes exist, for example at NIAB, Cambridge, UK. If these re‐synthesised bread wheats are made using *A. tauschii* accessions with a *Gli‐D2* locus containing low‐immunogenic alpha‐gliadins, the resulting hexaploid would be safer for CD patients (Smulders *et al*., 2015). To this end, we need to identify *A. tauschii* accessions that contain gliadins with fewer CD epitopes.

Here we have screened the CD‐immunogenic potential of a diverse panel of *A. tauschii* accessions by deep sequencing of the N‐terminal region of alpha‐gliadin transcripts, which contains the repetitive domain with CD epitopes as described by Salentijn *et al*. (2013). Using the frequency of reads as a proxy for the level of gene expression, we estimated the occurrence of CD epitopes and the relative toxicity of *A*. *tauschii* accessions for CD patients. We also determined the occurrence of specific variants, including the 33‐mer, and whether safe gene variants exist. These results were related to the geographical region and genetic cluster to which the accessions belonged to determine the relative CD immunogenicity of subgroups of *A*. *tauschii* accessions and their potential for breeding safer wheat cultivars.

## RESULTS

### 454 RNA‐amplicon sequencing

Sequencing of amplified transcript fragments, representing variants of the first variable domain of alpha‐gliadin that were expressed in the wheat grain endosperm, was performed from the 3′‐end, directly entering the first repetitive domain, instead of the 5′‐end (i.e. entering the signal peptide first) as done in Salentijn *et al*. (2013) in tetraploid durum wheat. This resulted, at similar sequence depth, in double the number of usable reads for corresponding samples than obtained by Salentijn *et al*. (2013), with qualitatively similar cDNA sequences (Table S1 in the online Supporting Information).

### Diversity of unique alpha‐gliadin peptide variants

The reads were organised into ‘unique sequence clusters’ containing the cDNA sequences that were 100% similar and with a sequence depth of >20 reads. By translating the nucleotide sequences of these unique sequence clusters, ‘unique peptide variants’ (UPVs) were deduced (Table S2). Most of these peptide variants were represented by several different unique nucleotide sequences, so they may originate from different genes. As our analysis focused on the peptide variants and their characteristics, our results may therefore underestimate the number of different genes present in the genome and being expressed. Moreover, alpha‐gliadin genes that have an identical coding sequence may have different promoter regions, as found by Noma *et al*. (2016) who cloned all alpha‐gliadin genes from the hexaploid cultivar CS.

In developing grains of each accession, several alpha‐gliadin peptide variants were expressed (Table S3). Using a lower threshold of 0.1% of the reads for all peptide variants in a sample, an average of 66 variants were expressed in hexaploid bread wheat lines compared with 36 in the grains of diploid *A. tauschii*; however, the average number of abundant peptide variants (>5% of the total number of reads) was almost the same at six (ranging from five to seven) and five (ranging from three to seven). The number of peptide variants in the tetraploid *T. turgidum* accession Primadur was remarkably low at 14 variants (>0.1%), with four being abundant (>5%). All data are in Table S4.

In this study a total of 349 new alpha‐gliadin peptide variants were found, while 38 had been described before by Salentijn *et al*. (2013) in tetraploids (Table S3). Of these 387 peptide variants, 27 were present in both *T. aestivum* and *A. tauschii*, while 211 and 149 were unique to *T. aestivum* and *A. tauschii*, respectively. Salentijn *et al*. (2013) reported 171 unique peptide variants from 61 different *T. turgidum* accessions. Because of the sequencing technology used, these novel peptide variants included 62 short variants of only 50 amino acids in length. These were devoid of the immunodominant DQ2.5‐Glia‐α1a, DQ2.5‐Glia‐α1b, DQ2.5‐Glia‐α2 T‐cell epitopes, but some of them did contain the DQ2.5‐Glia‐α3 T‐cell epitope and/or the innate peptide p31‐43.

### Patterns of expressed gene variants

**Figure 1:**
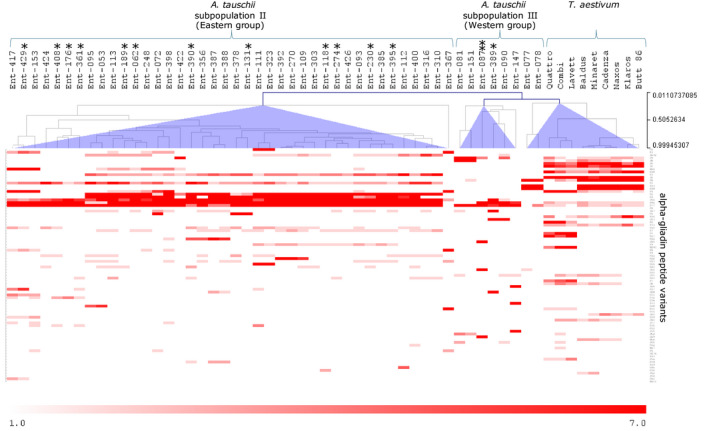
Alpha‐gliadin peptide variant expression profiles in grains of *Aegilops tauschii* (Ent lines) and *Triticum aestivum* accessions. The peptide variants were differentially abundant in the accessions. The accessions were clustered based on their peptide variant expression profile using hierarchical clustering with Spearman rank correlation with average linking. Purple triangles highlight the main clusters. Ent lines marked with * and ** contain peptide variants with the 33‐mer. *Variant J234. **Variant J2 and J29. Variant J2 was also found in all of the nine bread wheat accessions.

Hierarchical cluster analysis of the abundance of alpha‐gliadin peptide variants across the *T. aestivum* and *A. tauschii* accessions identified several groups of accessions with a similar peptide variant expression pattern, although some *A. tauschii* accessions had a unique expression pattern (Figure 1). The main difference in the pattern of gene expression was between the Eastern (group II) and Western (group III) *A*. *tauschii* accessions. The pattern of gene expression was quite similar for the group II accessions, which is consistent with this group having a lower level of genetic diversity (Jones *et al*., 2013; Wang *et al*., 2013).

The pattern of peptide variant expression in *A. tauschii* lines from subpopulations IIID and IIIE, and especially of the subpopulation IIID lines Ent‐077 and Ent‐078, was most similar to that observed in the bread wheat lines. This is in accordance with the suggestion of Jones *et al*. (2013) that, based on microsatellite data, *A. tauschii* from subpopulation III and, more specifically, subpopulations D and E, has the closest relationship to bread wheat.

### Epitopes present in the alpha‐gliadin peptides

The alpha‐gliadin peptide variants cover the region of alpha‐gliadins with the p31‐43 epitope responsible for the innate immune response, and the region with immunodominant DQ2.5‐Glia‐α1a, DQ2.5‐Glia‐α1b, DQ2.5‐Glia‐α2 and DQ2.5‐Glia‐α3 T cell epitopes. The peptide variants differ in epitope makeup and therefore in putative toxicity. All *A. tauschii* accessions contain several alpha‐gliadin peptide variants with multiple sets of DQ2.5‐Glia‐α T‐cell epitopes, but some accessions [Ent‐095 (group IIB), Ent‐422 (group IIC), Ent‐151 (group IIID), Ent‐081 (group IIID)] also contain a significant number (8–10%) of peptide variants that lack any of the canonical epitopes (peptide variant C4 and J9) (Table S5). Furthermore, several accessions contain peptide variants with only the DQ2.5‐Glia‐α3 T‐cell epitope, which is regarded as the least toxic of the three (Anderson *et al*., 2006). In one such accession, Ent‐389 (group IIB), 40% of the peptide variants only contain the DQ2.5‐Glia‐α3 T‐cell epitope (variant J13, J17 and J28 with relative abundances of 17%, 15% and 8%, respectively).

### Occurrence of the 33‐mer

The 33‐mer peptide with six overlapping CD epitopes, which is a unique feature of D genome alpha‐gliadins in bread wheat, was not found in *A. tauschii* in earlier studies (Ozuna *et al*., 2015; Huo *et al*., 2018) and these authors presumed that it may have developed after the hybridisation event. In our study, we ensured that we included *A. tauschii* accessions covering the whole distribution area of the species, i.e. the Eastern as well as the Western areas, as well as all subtypes within those regions. In our data we did find the 33‐mer in some of the *A*. *tauschii* accessions sequenced in this study, always as a small fraction of the transcripts. The 33‐mer was present in intact form in three alpha‐gliadin peptide variants: J2, J29 and J234. Variant J234 was found in 13 different *A. tauschii* accessions (12 of subpopulation II and one of subpopulation III; marked with * in Figure 1), always at low frequency (<0.5%). Variants J2 and J29 were only found in *A. tauschii* accession Ent‐087 (marked with ** in Figure 1) (subpopulation IIID) and were expressed at 0.05% and 6.8% of all transcripts in this accession, respectively. J2 was the only one that was found in both *A. tauschii* accession Ent‐087 and in all of the nine bread wheat accessions, in which it was more abundantly expressed (5–16% of all transcripts). In these nine hexaploids, ten other peptide variants (J60, J76, J89, J99, J208, J233, J234, J252, J273, J282) also contained the 33‐mer, but these were all present at low frequencies (maximum 0.7%). We have not detected these variants in our set of *A. tauschii* germplasm, which may be due to the limited number of *A. tauschii* accessions included that are from IIID.

### Estimated CD epitope load

The alpha‐gliadin peptide variants differ in CD epitope composition (Table S3). The frequency of DQ2.5‐Glia‐α1a, DQ2.5‐Glia‐α1b, DQ2.5‐Glia‐α2 and DQ2.5‐Glia‐α3 epitope sequences in the total transcripts of different accessions was estimated by (i) determining the epitope composition for each individual alpha‐gliadin peptide variant and (ii) taking the frequency of the individual peptide variants into account. Epitope variants of DQ2.5‐Glia‐α1a (PFPQLQLPF and PFPHLQLPY) and DQ2.5‐Glia‐α2 (FLPQLPYPQ) for which toxicity has been demonstrated (Mitea *et al*., 2010) were included in the estimation.

**Figure 2:**
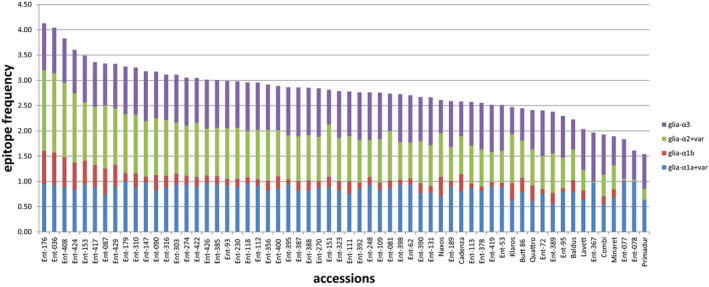
Accumulated frequency of different DQ2.5‐Glia‐alpha epitopes. Accumulated frequency of different DQ2.5‐Glia‐alpha epitopes in transcripts of different accessions: glia‐ α1a+var, DQ2.5‐Glia‐α1a (PFPQPQLPY) and its toxic variants DQ2.5‐Glia‐α1‐varT1 and‐varT2 (PFPQPQLPF and PFPHPQLPY); glia‐ α1b, DQ2.5‐Glia‐α1b (PYPQPQLPY); glia‐α2+var, DQ2.5‐Glia‐α2 (PQPQLPYPQ) and its toxic variant DQ2.5‐Glia‐α2‐varT1 (FLPQLPYPQ); glia‐α3, DQ2.5‐Glia‐α3 (FRPQQPYPQ).

In the alpha‐gliadin peptide variants of all *A. tauschii* and *T. aestivum* accessions a significant frequency of DQ2.5‐Glia‐α1a and DQ2.5‐Glia‐α3 epitopes was found, but all *T. aestivum* accessions had a lower frequency of these epitopes than most of the *A. tauschii* accessions (Figures 2 and S1).

Epitope DQ2.5‐Glia‐α1b was far less abundant and showed more variation in frequency among all accessions and was (almost) absent in a few *A. tauschii* accessions (Figure S1). DQ2.5‐Glia‐α2 frequencies were most variable in the alpha‐gliadin peptides and were high in some *A. tauschii* accessions and very low in some others.

The lowest total frequency of DQ2.5‐Glia‐α epitopes was found in *A. tauschii* accessions Ent‐077, Ent‐078 and Ent‐367 (Figure S1).

### Occurrence of the p31‐43 peptide

**Figure 3:**
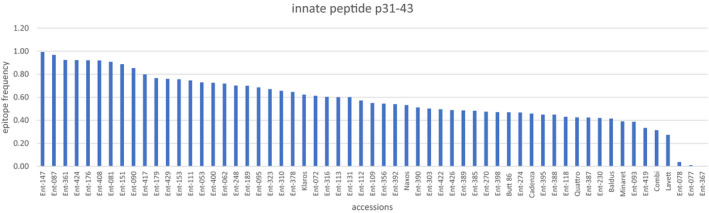
Relative frequencies of alpha‐gliadin peptide variants that contain the p31‐43 peptide in *Aegilops tauschii* and *Triticum aestivum* accessions.

The gliadin peptide p31‐43, which induces an innate immunity response in CD patients, is present in 32% of the peptide variants and its presence shows no correlation (*r* = 0.42) with the presence of DQ2.5‐Glia‐α epitopes. The *A. tauschii* accession Ent‐367 appears to be completely devoid of gliadin peptide p31‐43, whereas in Ent‐77 and Ent‐78 only a few peptide fragments contain it (Figure 3). These accessions also had a low frequency of DQ2.5‐Glia‐α epitopes.

## DISCUSSION

Deep amplicon sequencing of the first repetitive domain of alpha‐gliadin gene transcripts enables prediction of the (relative) CD toxicity of the alpha‐gliadin protein fraction in wheat. Based on the results reported here we analysed the translated protein expression patterns of *A*. *tauschii*. This indicated that the expression profile of the alpha‐gliadins was different between accessions from Western and Eastern clades of *A. tauschii*. Within each of these groups the expression profile varied among accessions. Both clades expressed many allelic variants that are not found in bread wheat, confirming and extending the results of Yan *et al*. (2003). In particular, the alpha‐gliadin peptide variants J11, C1 and C2 are abundantly present in several *A. tauschii lines* (up to 35%, 17% and 18%, respectively) but are not found in bread wheat.

Alpha‐gliadins containing the 33‐mer peptide are regarded as the most CD‐toxic variants in bread wheat. This αGlia‐33‐mer fragment is naturally formed in the human gastrointestinal tract by digestion with gastric and pancreatic enzymes, it binds well to DQ2 after deamidation by tissue transglutaminase (TG2), and it is recognised much more effectively by intestinal T‐cell lines than shorter peptides covering only the DQ2‐α‐I, ‐α‐II or ‐α‐III epitopes (Shan *et al*., 2002). The 33‐mer is present in the alpha‐gliadins of the D genome of bread wheat (chromosome 6D, locus Gli‐2D) (Molberg *et al*., 2005; Spaenij‐Dekking *et al*., 2005; Van Herpen *et al*., 2006; Mitea *et al*., 2010; Salentijn *et al*., 2013). Ozuna *et al*. (2015) and we therefore expected it to be present in *A. tauschii* accessions, but we did not find it in 22 alpha‐gliadin genes from three *A. tauschii* accessions and concluded that the 33‐mer may have evolved in the hexaploid. We screened a wide set of accessions covering the complete distribution area of the species, and found the 33‐mer in only a few of the *A. tauschii* accessions. Just one of the *A. tauschii* accessions included in our study (Ent‐087) expressed the 33‐mer at a significant frequency. This accession was also the only *A. tauschii* accession in our study that shared an identical peptide variant containing a 33‐mer with the hexaploid bread wheat accessions in this study. Accession Ent‐087 is from the IIID subpopulation, which is from the region close to the Caspian Sea that is considered to be where the species hybridisation that led to bread wheat took place (Dvorak *et al*., 1998). This is consistent with the notion that the D genome in bread wheat has a narrow genetic basis.

Overall, sufficient genetic variation is present in the alpha‐gliadins in *A. tauschii* to try to identify *A. tauschii* accessions with a reduced CD epitope load. When we screened our sequences for variation in CD epitopes, no *A. tauschii* accessions were found without the DQ2.5‐Glia‐α T cell epitope but some accessions do express alpha‐gliadin proteins that have no or a few epitopes. Almost all accessions express alpha‐gliadin peptide variants that contain only the DQ2.5‐Glia‐α3 T cell epitope, which is regarded as least toxic of the three DQ2.5 alpha‐gliadin epitopes. This T‐cell epitope has been shown to make only a minor contribution to the gluten‐induced T‐cell response in HLA‐DQ2+ CD (Anderson *et al*., 2006). These accessions can be used to generate less toxic synthetic hexaploid wheat (SHW) lines, by hybridising these accessions with tetraploid wheat accessions, which would form a good basis for of a breeding programme to generate hypoimmunogenic or even CD‐safe bread wheat (Jouanin *et al*., 2018, 2020b). The tetraploid wheat accessions may be selected based on genetic characteristics or agronomic performance.

We have screened *A. tauschii* accessions as we believe that finding differences among them is more efficient than screening SHWs. First, some *A. tauschii* accessions tend to be used frequently for SHWs and others not, because they have interesting phenotypes unrelated to gluten, such as disease resistance. Second, another potential problem is that the expression of gliadins (particularly novel variants) may be masked or changed in the hexaploid background, and this may depend on the genotype with which it is hybridised, so that a broad screening becomes more complicated when using SHW lines. This is why recent gene identification from wild relatives has focused directly on the progenitor species [e.g. in the cloning of resistance genes using R gene enrichment sequencing (AgRenSeq) in *A. tauschii* by Arora *et al*. (2019)].

Several of the studied *A. tauschii* accessions have already been used at NIAB for production of synthetic hexaploid wheat lines (Table S6). In such a programme, targeted gene editing with CRISPR/Cas9 could be included later on to edit the remaining epitopes or remove some alpha‐gliadins (Smulders *et al*., 2015; Jouanin *et al*., 2019), as *A. tauschii* cannot easily be transformed or regenerated. Alternatively, one could apply Tilling to improve a specific *A. tauschii* line (Rawat *et al*., 2018).

Whether gene expression of alpha‐gliadins is affected by polyploidisation cannot be deduced from modern bread wheat varieties, as they are the result of 10 000 years of selection and breeding after the allopolyploidisation event and the original D genome donor is no longer available. It can be studied, though, by comparing the gene expression of developing grains of particular *A. tauschii* accessions with those of the grains of the synthetic hexaploids produced by hybridising these accessions to a few tetraploid lines, when grown side by side. For the use of specific *A. tauschii* accessions for introgression of less toxic variants it will be imperative to compare their gene expression with those at the allohexaploid level. Such synthetic hexaploids are currently being made.

## EXPERIMENTAL PROCEDURES

### Plant material

A subset of 51 different *A. tauschii* accessions used by Jones *et al*. (2013) in their diversity study and selected to be representative of the D genome diversity in *A. tauschii*, was sampled at NIAB. We adapted the subgroup codes of Jones *et al*. (2013), who used group II for the Eastern clade of *A. tauschii* accessions [1E and 1W in Wang *et al*. (2013), L1 in Singh *et al*. (2019)] and group III for the Western clade [2E and 2W in Wang *et al*. (2013), L2 in Singh *et al*. (2019)]. Furthermore, a panel of nine different bread wheat (*T. aestivum*) varieties and one durum wheat (*T. turgidum*) variety was included (Table S7). For transcript sequencing, immature spikes were harvested, frozen in liquid nitrogen and stored at −80°C until RNA extraction.

### Extraction of RNA and cDNA synthesis

For each genotype, total RNA was extracted from wheat endosperm of a pool of five immature grains from a single spike at the milk to soft dough ripening stage. For some samples dry, mature grains were also used for total RNA isolation. The RNA was extracted according to Salentijn *et al*. (2013) but with a simple improvement to the RNA extraction protocol, consisting of removal of starch and polysaccharides prior to TRIzol extraction (Li and Trick, 2005). For this, 50–100 mg of fine‐ground grains was suspended in RNA extraction buffer (Li and Trick, 2005) and subjected to a chloroform/phenol extraction. The supernatant was then mixed with TRIzol and chloroform, and after TRIzol extraction the supernatant was further purified using the Qiagen RNeasy Plant Mini Kit (http://www.qiagen.com/). Total RNA was finally eluted from the RNeasy spin column using 50 µl of water. The RNA quality was checked using agarose gel electrophoresis and total RNA quantity was determined spectrophotometrically (NanoDrop ND1000, NanoDrop Products, http://www.thermofisher.com/). Prior to reverse transcription, traces of genomic DNA were removed by a DNaseI treatment using the Invitrogen DNase kit (http://www.thermofisher.com/). Subsequent cDNA synthesis and PCR amplification of alpha‐gliadin transcript fragments and pooling of PCR samples was performed exactly as described by Salentijn *et al*. (2013) using the same gene‐specific primers but with the unique 10 bp ID sequences included in the reverse primers.

### 454 sequencing and data analysis

Roche/454 amplicon sequencing and data analysis were performed essentially as described before (Salentijn *et al*., 2013). For this the signal peptide and the repetitive domain of alpha‐gliadin transcripts were amplified and sequenced. Sequencing was now started from the 3′‐end, directly entering the repetitive domain, instead of starting from the 5′‐end (signal peptide) as performed by Salentijn *et al*. (2013). On average 2184 reads per sample were obtained (minimum 199 reads, median 1632 reads, maximum 9409 reads). For some samples that yielded only a few hundred sequence reads this may give a bias towards the most abundant variants only. This was also found when we compared the abundance of gliadin transcripts in samples originating from dry grains with that of immature grains from the same genotype. Because RNA isolated from dry seed was of poorer quality, lower‐quality reads were obtained which represented the most abundant sequences that were found in transcripts from immature seeds (Table S8). For some samples replicate RNA extractions and sequencing from seeds of the same genotype were performed to show the reproducibility of the method (Table S8). After pre‐processing (as described in Salentijn *et al*., 2013) the transcript sequences were clustered using USEARCH v.4.0. First, all sequences were clustered at 100% homology, clusters were sorted according to the number of reads and for all clusters with more than 20 reads (a total of 280 571 sequences) the reads were then clustered at 99.5% homology in order to combine sequences from a single gene while allowing for typical 454 sequencing errors. The output of the pipeline consisted of the consensus cDNA sequences of these clusters (572 clusters in total), the deduced amino acid sequences (unique peptide fragments), the number of 454 reads per cluster per sample and the number of DQ2.5 CD epitopes in their non‐deamidated forms [DQ2.5‐Glia‐α1a (PFPQPQLPY) and its toxic variants DQ2.5‐Glia‐α1‐varT1 and‐varT2 (PFPQPQLPF and PFPHPQLPY), DQ2.5‐Glia‐α1b (PYPQPQLPY), DQ2.5‐Glia‐α2 (PQPQLPYPQ) and its toxic variant DQ2.5‐Glia‐α2‐varT1 (FLPQLPYPQ) and DQ2.5‐Glia‐α3 (FRPQQPYPQ)] and presence/absence of the innate peptide p31‐43 and the 33‐mer peptide. Several transcripts contain internal stop codons and can be regarded as pseudogenes. They were always present at low transcript abundance. If the stop codon was downstream of the DQ2.5 epitopes, these pseudogene transcripts were included in the epitope calling. Peptides in a sample were called based on the percentage of reads for that peptide relative to the total number of reads for all peptide variants in a sample. The call thresholds were 0.1% for rare variants and 5% for abundant transcripts.

## Author contributions

NG, LJWJG and MJMS initiated the research. NG and ARB provided the material. EMJS, JGS and SVG extracted the RNA and produced the libraries. JGS, EMJS, CC and DGE analysed the sequence data. CC performed the epitope predictions. JGS and MJMS wrote the manuscript, with revisions from the others. All authors read and approved the final version.

## Conflict of interest

The authors declare no conflict of interest.

## Supporting information


**Table S1.** Old versus new 454 approach.

**Table S2.** Clustered transcript sequence data and processing. Clustered transcript nucleotide sequences were translated into proteins and unique peptide variants were grouped.

**Table S3.** Unique peptide variants with epitope load.

**Table S4.** Unique peptide variants variant frequencies for each accession.

**Table S5.** Epitope frequency for each accession.

**Table S6.** List of *Aegilops tauschii* accessions captured in re‐synthesised hexaploid wheat (SHW). All SHW are available via the NIAB Breeder’s Toolkit (http://www.niab.com/pages/id/419/Breeders__Toolkit).

**Table S7.** Wheat accessions used. A list of the *Aegilops tauschii* lines studied (annotation from Jones *et al*., 2013) and the bread wheat and durum wheat lines included for comparison.

**Table S8.** Reproducibility of 454 sequencing.

**Figure S1.** Frequency of different DQ2.5‐Glia‐alpha epitopes in transcripts of different accessions.

## Data Availability

All relevant data on the peptides and epitopes can be found within the manuscript and its supporting materials.
