# Supplementary material for: Exploring the alpha‐gliadin locus: the 33‐mer peptide with six overlapping coeliac disease epitopes in Triticum aestivum is derived from a subgroup of Aegilops tauschii
Source: Plant J. 2021 Feb 19;106(1):86–94. doi: 10.1111/tpj.15147 (PMC8248119; doi:10.1111/tpj.15147)
Supplement: Supplementary file 7 — Table S7. Wheat accessions used. A list of the Aegilops tauschii lines studied (annotation from Jones et al., 2013) and the bread wheat and durum wheat lines included for comparison. [file TPJ-106-86-s008.docx]

Supplementary Table S7. *Aegilops tauschii* accessions captured in re-synthesised hexaploid wheat (SHW).

All SHW are available via the NIAB Breeder’s Toolkit (<http://www.niab.com/pages/id/419/Breeders__Toolkit>)

| *Aegilops tauschii* accession | NIAB Synthetic Hexaploid Wheat |
| --- | --- |
| Ent-077 | NIAB-SHW071 |
| Ent-078 | NIAB-SHW095 |
| Ent-081 | NIAB-SHW096 |
| Ent-087 | NIAB-SHW083 |
| Ent-090 | NIAB-SHW082 |
| Ent-118 | NIAB-SHW086 |
| Ent-270 | NIAB-SHW080 |
| Ent-310 | NIAB-SHW150 |
| Ent-389 | NIAB-SHW085 |
| Ent-392 | NIAB-SHW054 |
