# Supplementary material for: Exploring the alpha‐gliadin locus: the 33‐mer peptide with six overlapping coeliac disease epitopes in Triticum aestivum is derived from a subgroup of Aegilops tauschii
Source: Plant J. 2021 Feb 19;106(1):86–94. doi: 10.1111/tpj.15147 (PMC8248119; doi:10.1111/tpj.15147)

Suppl figure 1. Frequency of different DQ2.5-Glia-alpha epitopes in transcripts of different accessions


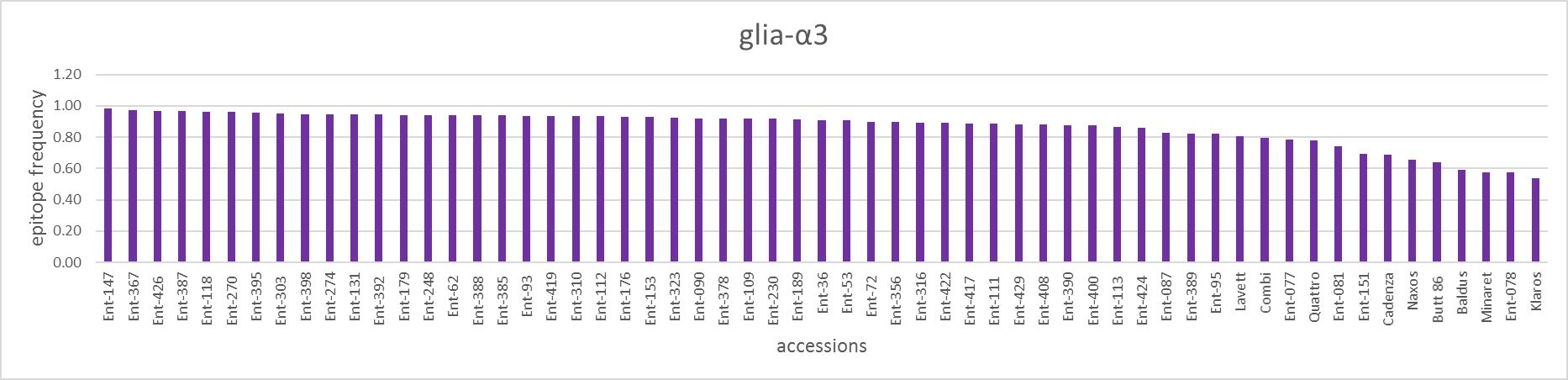

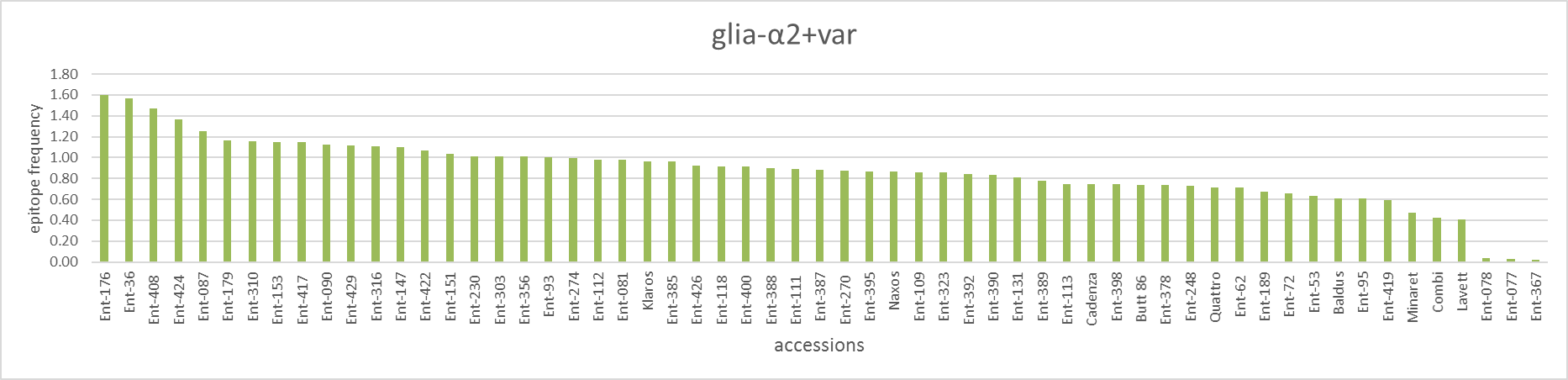

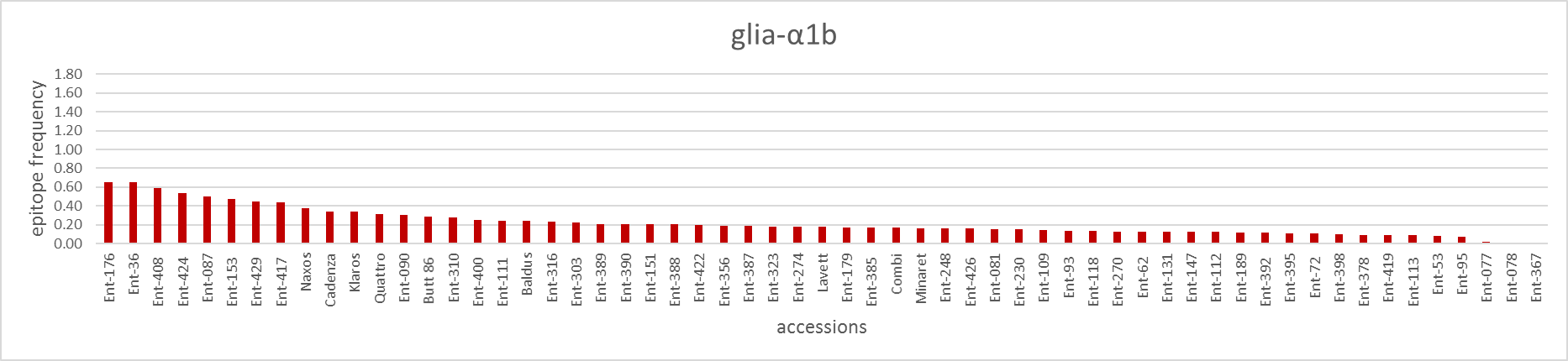

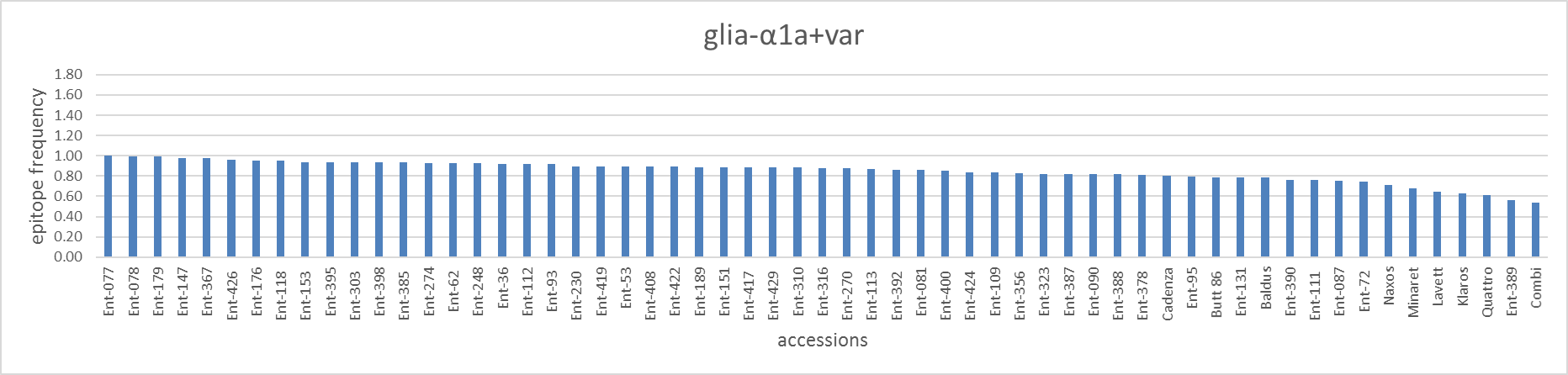

Supplement: Supplementary file 9 — Figure S1. Frequency of different DQ2.5‐Glia‐alpha epitopes in transcripts of different accessions. [file TPJ-106-86-s005.docx]
